# It's Not Always Gynecomastia: A Case of Diffuse Large B-Cell Lymphoma Presenting as Breast Mass in a Male Patient

**DOI:** 10.7759/cureus.12944

**Published:** 2021-01-27

**Authors:** Amie Leon, Ashley R Way, Smita Sharma, Haley Letter

**Affiliations:** 1 Radiology, University of Florida College of Medicine – Jacksonville, Jacksonville, USA

**Keywords:** large b-cell breast lymphoma, non-hodgkin’s lymphomas, eccentric location, male breast cancers

## Abstract

We report a case of diffuse large B-cell breast lymphoma that presented as a palpable breast lump in a male patient evaluated with digital mammography and targeted breast ultrasound (US) but ultimately confirmed by US-guided core needle biopsy. We will discuss the appropriate workup algorithm for a palpable breast lump in a male patient as outlined by the American College of Radiology (ACR) Appropriateness Criteria. While gynecomastia is the most common reason for a new palpable breast lump in a male patient, male breast cancer (including lymphoma and metastasis) can have a similar appearance on imaging. Our goal is to provide clarity on proper radiographic assessment protocols and imaging features of palpable breast masses in men by emphasizing the role of anatomical location and symmetry in distinguishing it from more common causes, such as gynecomastia, in future diagnostic imaging scenarios.

## Introduction

Lymphoma describes a neoplasm of white blood cells, natural killer (NK) cells, B-cell, or T-cell lymphocytes. Lymphomas may arise anywhere along the lymphatic system including lymph nodes (intranodal diseases) and in the lymphatic vessels or network (extranodal diseases) [1]. These small vessels form the lymphatic system, or ‘immunological highway,’ which serves to join various lymphatic organs (spleen, tonsils, thymus, and lymph nodes) facilitating the transportation of immunological substrates to the bloodstream in order to fight off infections. The two main forms of lymphoma are Hodgkin lymphoma and non-Hodgkin lymphoma, the latter of which is more common and encompasses over 90 cancer subtypes [1,2].

Diffuse large B-cell lymphoma (DLBCL) is one of several types of non-Hodgkin lymphomas (NHL). DLBCL represents 31% of NHLs, is composed of mature B-cells, and is the most common histological NHL subtype [3]. Recent statistics published by the National Institutes of Health (NIH), through the Cancer Institute’s Surveillance, Epidemiology, and End Results Program (SEER), reported an incidence of 5.6/100,000 and a death rate of 1.8/100,000 in men and women per year in the United States, based on age-adjusted rates from 2013 to 2017 cases and deaths [4]. The overall five-year relative survival rate is 63.8% and is maintained across age, sex, and race. However, the largest cohort (34%) is diagnosed as Stage III. This stage at diagnosis impacts a patient’s five-year survival rate. Literature reports 73.3% if diagnosed at/in Stage I, 72.7% at Stage II, 63.7% at Stage III, 52.7% at Stage IV, and 57.7% for unstaged/unknown. DLBCL is a treatable disease, often responding to systemic chemotherapy and concurrent or adjuvant radiotherapy [4].

In the male population, the prevalence of any breast lymphoma is low as men represent less than 1% of total breast malignancies [1]. Breast lymphomas are classified as either primary breast lymphoma (PBL) or secondary breast lymphoma (SBL) [5]. PBL implies that the breast mass is the first diagnosis of lymphoma with no other evidence of lymphoma at the time of diagnosis. Few studies have investigated clinical presentation, radiographic traits, as well as the prevalence of PBL, but there lacks a distinction in males. A case study in 2004 by Mpallas et al. summarized details from 24 previous cases in men and discussed the commonality of unilateral (85%) versus bilateral presentation of PBL, such as in their case on a 67-year-old male [6]. A retrospective study by Surov et al. evaluated 36 patients (35 females, one male), with a median age of 65 years, and reported a prevalence of B-cell lymphoma in 94% of the cohort [7]. Another study by Zhang et al. had similar findings of predominantly B-cell lymphoma (82.8%) in a retrospective study of 29 patients (28 females, one male) [8].

Albeit rare, no single and/or multi-institutional studies were found illustrating the epidemiology, pathophysiology, diagnosis, and/or clinical history/outcomes of PBL in male cohorts. Currently, there are fewer than 30 reported case studies on DLBCL in men presenting with either PBL or SBL. Available information regarding PBL is surmised from female cohorts, although specific case studies do address the trends of PBL in men, reviewing presentation at diagnosis as well as commenting on the nonspecific features found on various imaging modalities.

## Case presentation

**Figure 1:**
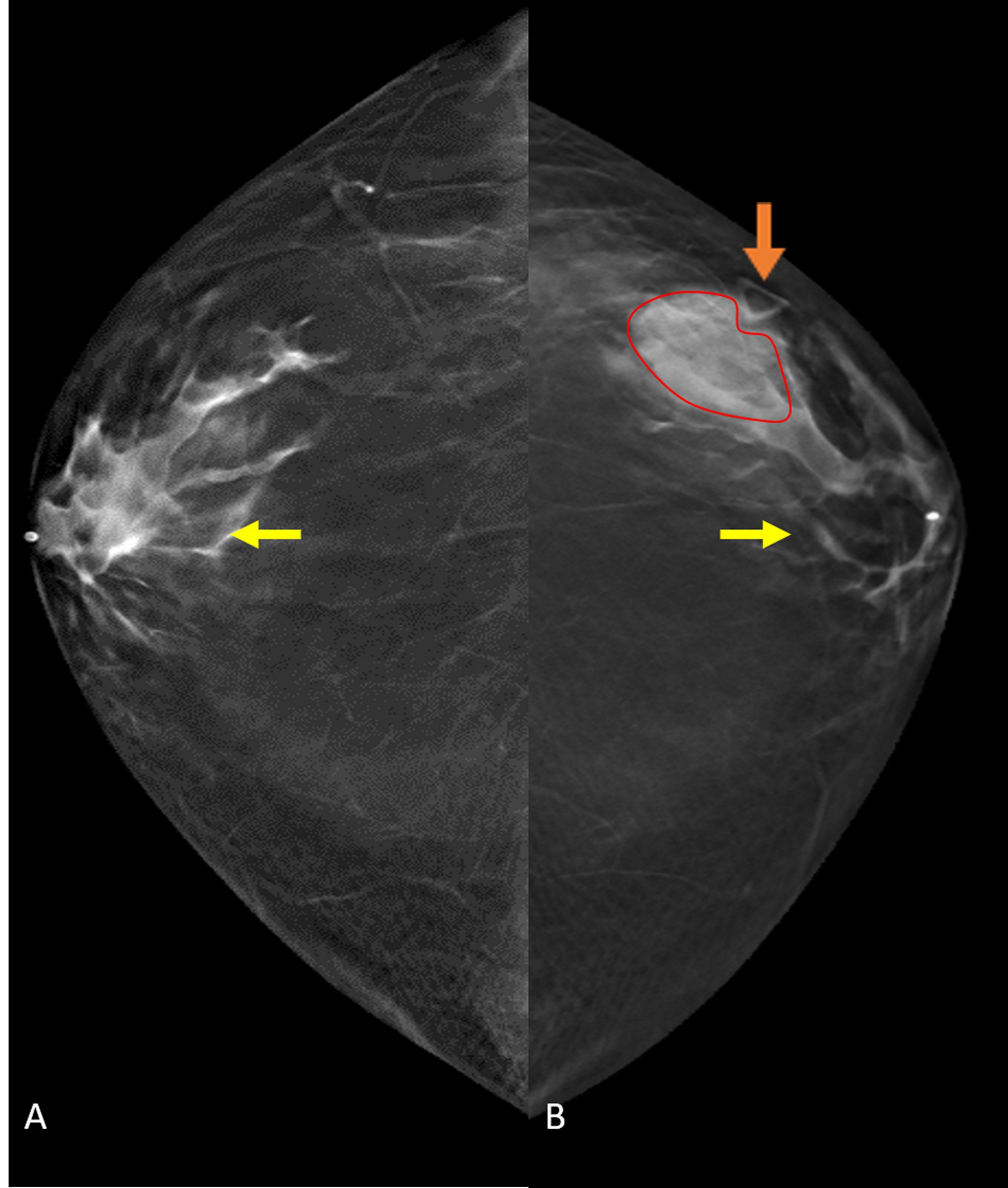
Bilateral CC diagnostic mammogram Images (A) and (B) demonstrate retro-areolar, flame-shaped, bilateral densities (yellow arrows), diagnostic of gynecomastia. However, in the outer left breast (B), there is a palpable triangular marker (orange arrow) overlaying a high density, irregular mass (red outline), eccentric to the nipple and atypical of gynecomastia, warranting further evaluation. CC, Craniocaudal.

**Figure 2:**
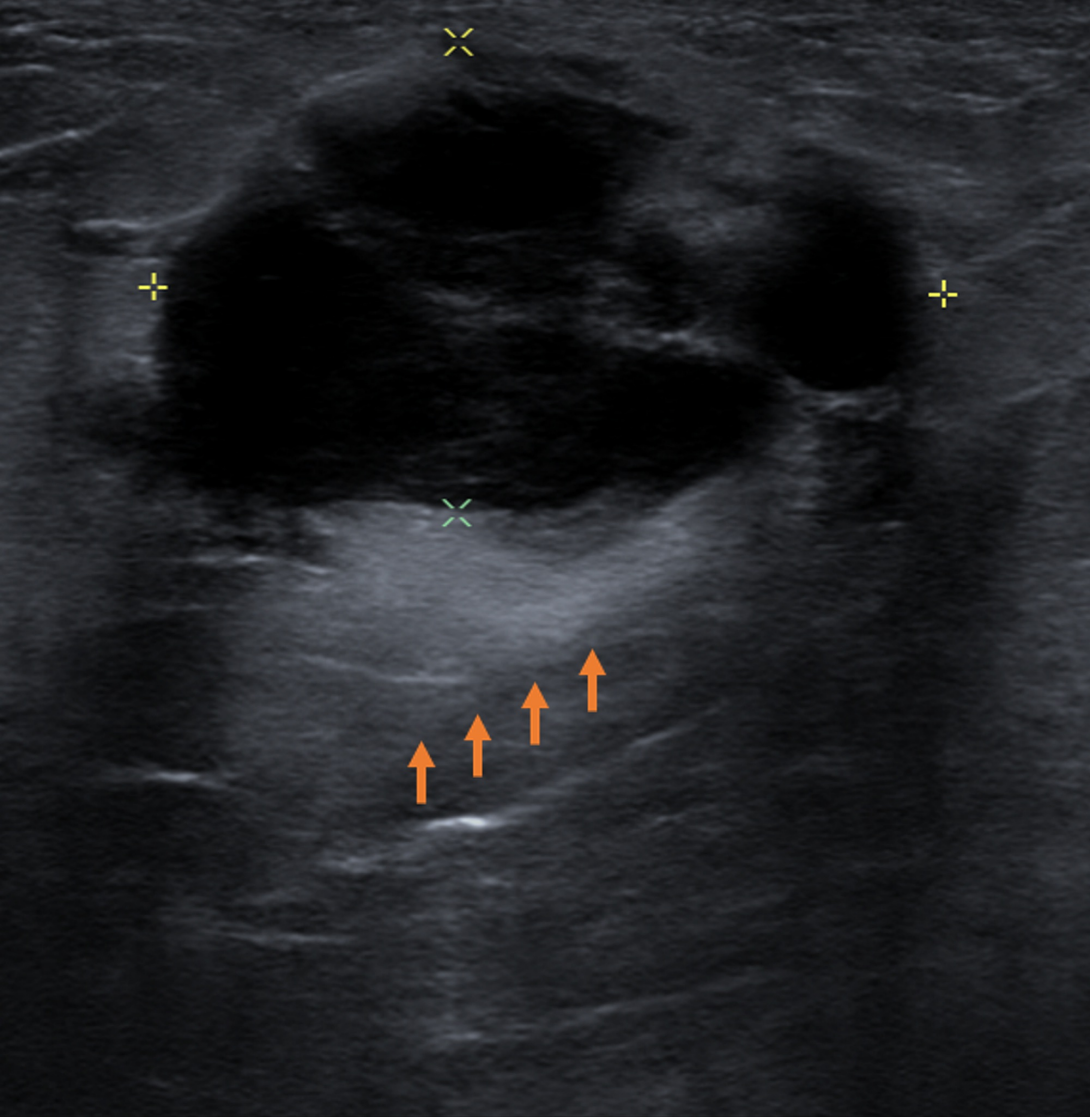
Targeted ultrasound of the left breast Targeted ultrasound of the left breasts demonstrates a complex solid and cystic mass at the 3:00 position, 5 cm from the nipple. There is a posterior acoustic enhancement (orange arrows). This should not be confused with a "complicated cyst." Complex solid and cystic masses should be biopsied (BI-RADS 4). BI-RADS, Breast Imaging Reporting and Database System score.

A 61-year-old male with no significant past medical history presented to his primary care physician reporting a one-week history of a non-tender palpable left breast mass. The patient denied fever, chills, night sweats, or weight loss. Physical exam was significant for a 2-cm firm, mobile mass, within the left outer breast. He was referred for breast imaging and underwent bilateral diagnostic mammogram with craniocaudal (CC) and mediolateral oblique (MLO) views. Mammography revealed an outer, mid-left breast focal asymmetry measuring 3.5 cm and located eccentric to the nipple, with no associated nipple retraction or skin thickening (Figure 1). Given the location of the focal asymmetry, targeted left breast ultrasound (US) was performed. This demonstrated a complex solid and cystic mass (3 cm x 1.9 cm x 3.3 cm) with thick internal septations and mild peri-lesion edema (Figure 2). Fine-needle aspiration (FNA)/core needle biopsy was recommended and was performed using US guidance. Pathology revealed c-MYC-positive DLBCL, activated B-cell type.

**Figure 3:**
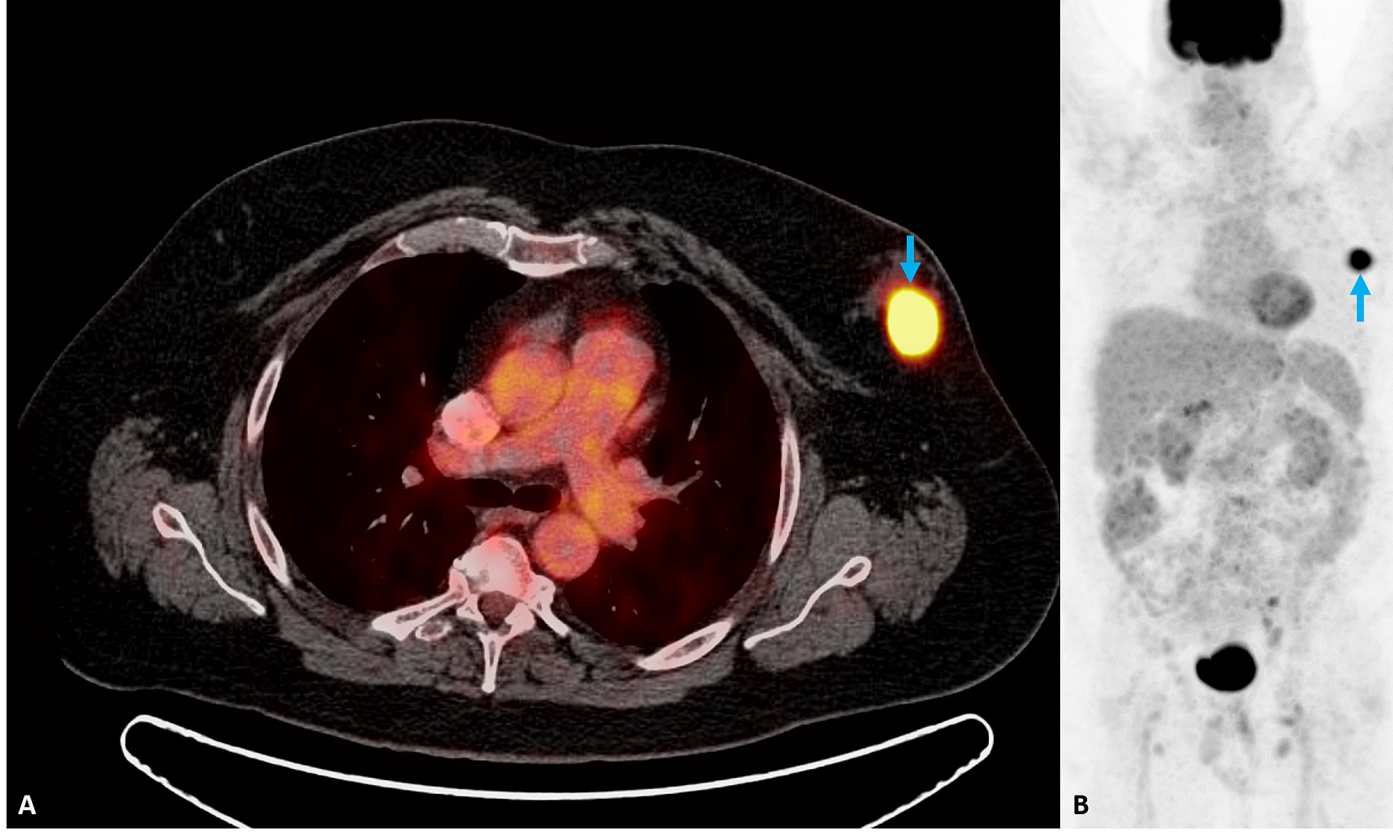
Pelvic PET/CT After initial diagnosis, further evaluation by (A) positron emission tomography/computed tomography (PET/CT) revealed a hypermetabolic area in the same location as the palpable nodule in question (blue arrow). PET imaging alone (B) revealing a hypermetabolic left breast mass (blue arrow).

**Figure 4:**
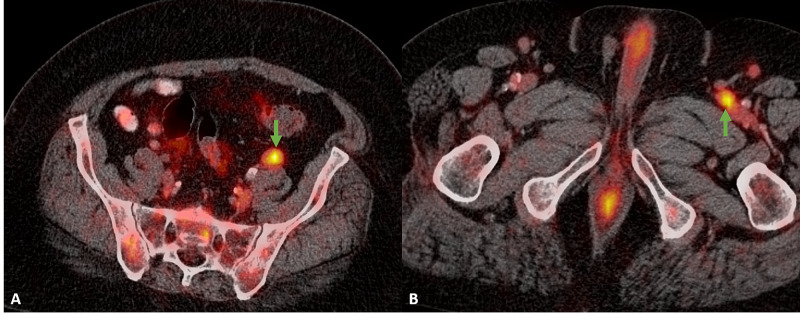
Pelvic PET/CT Images (A) and (B) demonstrate bilateral hypermetabolic inguinal lymph nodes (green arrows). PET/CT, Positron emission tomography/computed tomography.

Following the diagnosis of lymphoma, the patient underwent positron emission tomography/computed tomography (PET/CT) for complete staging. PET imaging showed a hypermetabolic left breast mass with a maximum SUV of 17.9 (Figure 3). Below the diaphragm, there were bilateral hypermetabolic iliac and inguinal lymph nodes (Figure 4). There was no hypermetabolic adenopathy above the diaphragm and no splenic involvement. Bone marrow biopsy revealed bone marrow involvement. The patient successfully underwent six cycles of chemotherapy and is now in remission.

## Discussion

Lymphoma of the breast is a rare presentation of NHL, with a reported prevalence of 0.4%-0.7% [1]. Patients presenting with PBL often report findings of a painless, palpable, unilateral, or bilateral (5%-11%) breast mass that are typically discovered upon self-examination or daily hygienic routine. PBL may also be found incidentally on routine imaging (12%) [1,9,10]. These masses are frequently located in the upper outer quadrants of the breast with some patients presenting concurrently with palpable masses in their ipsilateral axilla [11]. This is due to the anatomic location and drainage of the breast lymphatics, which are generally located concentrically to the areola [12].

Although anatomic variation does exist, the majority of lymph draining laterally to the anterior axillary/pectoral group of lymph nodes (78.4%) and medially to the internal thoracic lymph nodes (21.9%) contributes to this presentation [12]. DLBCL of the breast associated with nipple retraction, edema, erythema, skin thickening, or tender nodules is very rare; all of which are indications of inflammatory cancers or T-cell lymphoma [13]. Other symptoms associated with DLBCL include loss of appetite, fatigue, fever, itchiness, night sweats, and/or loss of 10% or more body weight over six months. The presence and severity of B symptoms are indicative of more advanced disease, are of higher staging, and are an indicator of poor prognosis [2,3,14].

These are important anatomical and clinical clues for distinguishing DLBCL of the breast from commonly presenting breast masses in men, which should be considered in the development of differential diagnosis. The most common benign cause of a palpable breast mass in a man is gynecomastia, but other benign causes may include epidermal inclusion cysts, lipoma, posttraumatic hematoma or fat necrosis, and pseudogynecomastia. Malignant variants of palpable breast masses include invasive ductal carcinoma, invasive lobular carcinoma, papillary carcinoma, and/or malignancy [13,14]. A summary of referenced imaging characteristics of common benign and malignant variants of palpable breast masses is provided in Table 1 [13,14].

**Table 1:** List of common benign and malignant variants of palpable breast masses and their associated imaging characteristics References for this table include [10-14]. DDX, Differential diagnosis; PMH, past medical history; PE, physical exam; w/, with.

DDx Breast Mass	Patient Presentation	Associated Features	Imaging Characteristics	
Benign			Mammography	Ultrasound
Gynecomastia. Note: Nodular, symptoms <1 year; dendritic, symptoms >1 year; diffuse, result of high-dose estrogen/exogenous hormones	Peri-pubertal and men over 50 y.o. with unilateral or bilateral breast tenderness. PE: Soft, compressible, mobile central subareolar mass w/ pain in early phase. Note: Breast enlargement without subareolar mass typical of pseudogynecomastia.	Proliferation of ductal and stromal elements. Note: Causes include drug-related, hormonal (estrogen/androgen imbalance), idiopathic, physiologic, or systemic disease.	Nodular: Fan-shaped subareolar density w/ subcutaneous fat blending. Dendritic; flame-shaped subareolar density w/ finger-like projections into fat; diffuse glandular; bilateral heterogeneously dense breasts; pseudogynecomastia; increased lucent subareolar fat.	Subareolar, hypoechoic, hypervascular, fan- to disk-shaped area.
Lipoma	PE: Slow growing and soft, non-tender, mobile, palpable subcutaneous mass.	Mature fat cells lacking malignant potential.	Encapsulated oval fat density.	Variable, hyperechoic w/o internal flow.
Angiolipoma	PE: Mobile, tender, palpable mass.	Mature fat cells and vessels.	Mixed fat and soft tissue density.	Note: Initial presentation warrants imaging escalation and is not usually imaged with ultrasound.
Lymph node	PE: Soft, rubbery, or firm mass typically in upper outer quadrant.	Normal lymph node.	Reniform circumscribed mass w/ dense outer cortex and lucent fatty central hilum.	Homogenous thin cortex (<2 mm) and echogenic fatty hilum.
Posttraumatic changes	PMH: Trauma or coagulopathy. PE: Irregular to well-defined mass.	Fat necrosis, hematoma.	Peripherally calcified oil cysts, fat-fluid levels, or dystrophic calcifications.	Absence of internal vascularity.
Sebaceous/epidermal inclusion cysts	PE: Skin findings of erythema, edema, tenderness, and pain.	Obstructed sebaceous gland, obstructed hair follicle/secondary skin trauma.	Oval, circumscribed, dense, and superficial mass.	Claw sign, sinus tract from mass to skin.
Hemangioma	PE: Vascular lesions.	Mass of vascular tissue. Note: Biopsy for exclusion of angiosarcoma.	Oval solid mass w/ circumscribed margins.	Venous flow on Doppler suggests venous malformation.
Subareolar abscess	PE: Nipple swelling, erythema, discharge, and pain.	Chronic ductal obstruction leading to inflammation and infection.	Ill-defined subareolar mass with surrounding trabecular thickening.	Complicated fluid collection with peripheral hyperemia.
Papilloma	PE: Palpable nipple mass w/ or w/o discharge.	Epithelial proliferation with fibrovascular core.	Dense, circumscribed, retro-areolar mass.	Intraluminal mass w/in dilated duct.
Malignant				
Invasive ductal carcinoma (DCIS)	PE: Small hardened mass, skin thickening, nipple retraction, and axillary lymphadenopathy.	Lacking specific features of tubular, mucinous, or micropapillary morphologies.	High density, round, oval, or irregular subareolar mass w/ possible circumscribed, indistinct, speculated, or microlobulated margins.	Solid, hypoechoic mass w/ irregular shape and possible speculated, angular, or micro-lobulated margins. Eccentrically located to nipple.
Papillary carcinoma	Note: 2:1 presentation men to women.	Lack myoepithelial cell layer along vascular core ranging from low-to-high grade pleomorphism.	Subareolar mass w/ possible circumscribed, irregular, or speculated margins.	In situ; cyst or dilated duct w/ complex mixed cystic and solid mass (murla nodule or papillary projection).
Lymphoma note: May be primary or secondary	PE: Painless soft, firm, or rubbery palpable breast mass eccentric to nipple. Note: +/- associated B symptoms.	Majority non-Hodgkin B-cell. Note: Solid, trabecular, or diffuse architecture.	Circumscribed mass eccentric to nipple.	Circumscribed to irregular, hypoechoic, solid mass.
Metastasis	Usually multiple masses, bilateral > unilateral; can be solitary.	Common: Melanoma, lymphoma, lung, ovarian.	Round, circumscribed.	Circumscribed mass, variable echogenicity. Usually increased vascularity.

According to the American College of Radiology (ACR) Appropriateness Criteria, bilateral diagnostic mammogram with or without digital breast tomosynthesis (DBT) is the initial step in evaluating a male patient with a palpable lump. The rationale for using mammography as the first-line imaging tool is that gynecomastia has a very classic mammographic appearance but can often appear indeterminate or suspicious on the targeted US. Given that gynecomastia is by far the most common reason for a breast lump in a male patient, beginning with mammography obviates the need for further imaging (i.e., targeted breast US) in the majority of cases [13]. Gynecomastia presents as a mass or focal asymmetry located directly behind the nipple-areolar complex. It can be unilateral and asymmetric, but the key to its diagnosis is the retro-areolar location. Any mass or asymmetry that is eccentric to the nipple should be further investigated with targeted breast US. Unless a simple cyst is discovered on the US, core biopsy is often necessary for definitive diagnosis of an eccentrically located mass or asymmetry in a male patient [12-14]. 

On mammography, most lymphomas are either circumscribed or indistinct masses, classically without calcifications, spiculation, or architectural distortion. Occasionally, there is a diffuse increase in parenchymal density without a discrete mass. US findings are also nonspecific, but often these masses are heterogeneous, hyperechoic, or complex cystic and solid, such as in our case example. In a similar clinical scenario, posterior acoustic enhancement is common [5,11]. 

Biopsy is performed using US guidance, and core biopsy is preferred over fine-needle aspiration. The biopsy is evaluated by the surgical pathologist for immunophenotyping, in order to identify the cancer cell type, cell proteins, or any changes in genes. This includes an immunohistochemical panel (IHC) with or without flow cytometry, fluorescence in situ hybridization (FISH) used for initial karyotyping for MYC rearrangement, which if found positive is further karyotyped for BCL2 and BCL6 rearrangements. Common patterns of DLBCL proteins include the presence of CD20 and CD45 but the absence of CD3 [2,15].

Following tissue diagnosis of lymphoma, PET-CT is usually performed for complete staging. Lymphoma is usually very hypermetabolic, and breast lymphoma is no exception.

Treatment for breast lymphoma is based upon the overall staging of the lymphoma and generally requires a combination of chemotherapy, surgery, and radiation therapy. Patients with Stage I or II disease are given first-line chemotherapy regimen, which includes RCHOP (rituximab, cyclophosphamide, doxorubicin, vincristine, prednisone) with or without adjuvant radiation therapy. Patients with Stage III or IV disease are suggested for a clinical trial or RCHOP with interim restaging after two to five cycles and escalation based on residual disease [2,5,9].

## Conclusions

Imaging features of breast lymphoma are non-specific, usually presenting as a mass or focal asymmetry on a mammogram located eccentric to the nipple, which helps distinguish it from gynecomastia. Given these nonspecific imaging findings, a wide differential diagnosis should be considered. This includes other more common causes of malignancy in the male breast including infiltrating ductal carcinoma and metastasis. Tissue sampling is ultimately needed to secure the diagnosis. Ordering physicians need to be aware that nonspecific findings do not necessarily rule out lymphoma as a possibility.
